# Supplementary material for: Salivary Complaints in Burning Mouth Syndrome: A Cross Sectional Study on 500 Patients
Source: J Clin Med. 2023 Aug 26;12(17):5561. doi: 10.3390/jcm12175561 (PMC10488611; doi:10.3390/jcm12175561)
Supplement: Supplementary file 1 [file jcm-12-05561-s001.zip › jcm-2532300-supplementary.pdf]

Supplementary Table S1: Clarification for Not meeting inclusion/ exclusion criteria (*n* = 49)

Number of patients	Reason for exclusion
9	Type 2 diabetes
5	Diagnosis of hypothyroidism and initiation of replacement therapy for less than 3 months
6	The hematological investigations showed sideropenia
3	Mildly positive ANA antibodies
3	The patients has changed antihypertensive medication for less than 3 months
4	Symptoms remission after challenge-dechallenge-rechallenge test
6	The patients report the onset of symptoms for less than three months
3	Patients under treatment with antidepressants
2	Patients with recent diagnosis of neoplastic pathology
1	Patient with facial nerve palsy (hemiparesis)
5	Patients diagnosed with oral lichen planus
2	Patients with a previous diagnosis of Obstructive Sleep Apnea Syndrome (OSAS)
